# High-energy mid-infrared sub-cycle pulse synthesis from a parametric amplifier

**DOI:** 10.1038/s41467-017-00193-4

**Published:** 2017-07-26

**Authors:** Houkun Liang, Peter Krogen, Zhou Wang, Hyunwook Park, Tobias Kroh, Kevin Zawilski, Peter Schunemann, Jeffrey Moses, Louis F. DiMauro, Franz X. Kärtner, Kyung-Han Hong

**Affiliations:** 1 Department of Electrical Engineering and Computer Science and Research Laboratory of Electronics, Massachusetts Institute of Technology (MIT), Cambridge, Massachusetts 02139 USA; 2 Singapore Institute of Manufacturing Technology, 2 Fusionopolis Way, Singapore, 138634 Singapore; 3 Department of Physics, The Ohio State University, Columbus, Ohio 43210 USA; 4 Center for Free-Electron Laser Science, DESY and Department of Physics, University of Hamburg, 22607 Hamburg, Germany; 5 BAE System, MER15-1813, P.O. Box 868, Nashua, New Hampshire 03061 USA; 6 School of Applied and Engineering Physics, Cornell University, Ithaca, New York 14853 USA; 7 The Hamburg Center for Ultrafast Imaging, Luruper Chaussee 149, 22761 Hamburg, Germany

## Abstract

High-energy phase-stable sub-cycle mid-infrared pulses can provide unique opportunities to explore phase-sensitive strong-field light–matter interactions in atoms, molecules and solids. At the mid-infrared wavelength, the Keldysh parameter could be much smaller than unity even at relatively modest laser intensities, enabling the study of the strong-field sub-cycle electron dynamics in solids without damage. Here we report a high-energy sub-cycle pulse synthesiser based on a mid-infrared optical parametric amplifier and its application to high-harmonic generation in solids. The signal and idler combined spectrum spans from 2.5 to 9.0 µm. We coherently synthesise the passively carrier-envelope phase-stable signal and idler pulses to generate 33 μJ, 0.88-cycle, multi-gigawatt pulses centred at ~4.2 μm, which is further energy scalable. The mid-infrared sub-cycle pulse is used for driving high-harmonic generation in thin silicon samples, producing harmonics up to ~19th order with a continuous spectral coverage due to the isolated emission by the sub-cycle driver.

## Abstract (web summary)

Stable sub-cycle pulses in the mid-infrared region allow damage-free investigation of electron dynamics in solids. Here, the authors develop a suitable source to this end which is based on an optical parametric amplifier.

## Introduction

Generation of high-energy, few-cycle mid-infrared (mid-IR) pulses has progressed markedly over the last decade, driven by a number of applications, such as coherent soft X-ray high-harmonic generation (HHG)^1–3^, incoherent hard X-ray generation in laser-induced plasmas^4^, sub-femtosecond electron emission^5^, two-dimensional infrared spectroscopy^6^ and time-resolved imaging of molecular structures^7^. The use of a carrier-envelope phase (CEP)-stable single-cycle or even sub-cycle pulse can inherently isolate the electron dynamics in the strong-field interactions^8, 9^. Therefore, high-energy, CEP-stable, sub-cycle mid-IR pulses can be a very unique tool for investigating ultrafast dynamics of strong-field interactions in solids and gases. Some examples are the sub-cycle control of electron motions via HHG in solids^10–13^, the sub-cycle electron tunnelling in nano-devices^14, 15^, the generation of isolated attosecond^16^ or even zeptosecond X-ray pulses^17^, controlling strong-field molecular ionisation and dissociation^18^, steering the atomic-scale motion of electrons^8^, and sub-femtosecond control and metrology of bound-electron dynamics in atoms^9^. In particular, the studies on the strong-field interactions in solids and nano-structures are opening a great opportunity towards petahertz electronics^19, 20^. Recently, intensive effort has been made to generate few-cycle mid-IR pulses using several techniques, such as optical parametric amplifier (OPA)^21, 22^, laser filamentation^23^ and difference-frequency generation (DFG)^24^. Adiabatic difference frequency generation has produced few-μJ, shapeable, single-cycle mid-IR pulses^25^, though the technique’s scalability to the sub-mJ level is not obvious and CEP stability has not yet been demonstrated. Self-compression after spectral broadening in dielectric materials^26–29^ has also been used for generating sub-two-cycle mid-IR pulses. Regarding to the generation of mid-IR sub-cycle pulses, four-wave mixing through filamentation in a gas^30^ and a technique that cascades DFG, spectral broadening, and chirp-compensation^31^ have been demonstrated. However, the former gives low pulse energy (~0.5 μJ)^32^ and limited energy scalability as well as conical emission, while the latter has unknown CEP stability due to the complex nonlinear processes involved besides the low energy (~1 μJ). These impose limitations in the applications to strong-field light–matter interactions. On the other hand, in the visible to short-wavelength IR range, high-energy sub-cycle pulses have been demonstrated using pulse synthesisers^8, 9, 33, 34^. Greater-than-octave-spanning spectra are generated and amplified in different spectral bands. After the phase management of individual bands, the multi-colour pulses are coherently combined to form a sub-cycle pulse. The main challenge of this approach is the complexity of the system because sophisticated phase control and stabilisation have to be implemented for eliminating the relative timing and phase jitters from the individual bands.

Coherent synthesis between the signal and idler of a type-I collinear OPA in a passive way is an intriguing alternative for sub-cycle pulse generation. With near-degenerate signal and idler wavelengths, the total OPA bandwidth can be huge in a collinear geometry when the group velocity dispersion at the degenerate wavelength is small^35^. The signal and idler pulses are tightly synchronised in an OPA by nature. However, this method is challenging in the visible/near-IR range for several reasons. First, the dispersion over multi-octave bandwidths is very large, such that the signal and idler pulses will require post-compression. Second, the signal and idler need to have a stable relative CEP, which can only be achieved when both the pump and signal pulses are CEP stable, as their phase difference is transferred to the idler CEP. In the visible and near-IR ranges, such schemes usually require active CEP stabilisation. In contrast, in the mid-IR, the dispersion of OPA media can be very low within the transmission window, which enables the direct synthesis of the signal and idler pulses without post-compression. Moreover, in the scheme described below, the 2.1-µm pump laser itself is a passively CEP-stabilised, optical parametric chirped-pulse amplifier (OPCPA), and its pulse width is short enough to pump a white-light generation (WLG) stage and generate an octave-spanning CEP-stable signal pulse for a mid-IR OPA.

Here we present a multi-gigawatt (GW) sub-cycle mid-IR pulse synthesiser based on in-line multiplexing of the signal and idler pulses from an OPA. Furthermore, we demonstrate HHG in silicon samples up to ~19th order and observed a continuous spectrum due to the sub-cycle driver pulses. The signal and idler pulses covering 2.5–4.4 µm and 4.4–9.0 µm, respectively, are synthesised automatically in the 2.1-μm pumped collinear type-I OPA, due to the minimal dispersion and temporal walk-off of a thin CdSiP_2_ (CSP) crystal, which also supports a phase-matching bandwidth greater than one octave at the idler wavelength. The CEP stability of both signal and idler pulses is ensured with passive stabilisation, as confirmed by the *f*-3*f* spectral interferometry measurements over more than 6 min. Synthesised pulses with 33 µJ energy and multi-GW peak power having 12.4 fs full-width at half maximum (FWHM) duration, characterised using a cross-correlation frequency-resolved optical gating (XFROG) device, are produced and used for driving HHG in thin silicon samples. Our demonstration offers an energy-scalable and technically simple platform of laser sources generating CEP-stable sub-cycle pulses in the whole midwave-infrared region for investigating isolated phase-sensitive strong-field interactions in solids and gases^8, 9, 16, 17^.

## Results

### Experimental setup of mid-IR OPA

**Fig. 1:**
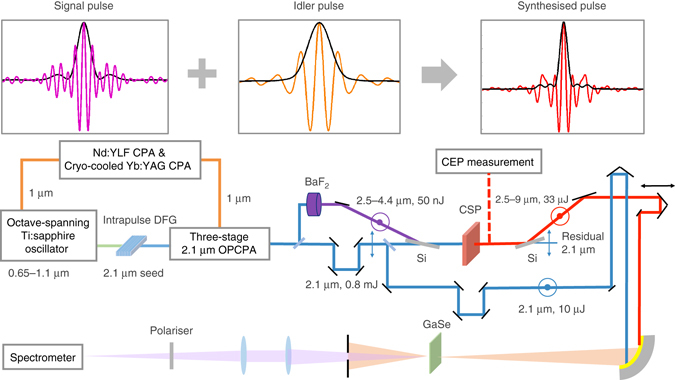
Schematic of the high-energy phase-stable sub-cycle mid-infrared optical parametric amplifier. *CEP* carrier-envelope phase, *CPA* chirped-pulse amplifier, *OPCPA* optical parametric chirped-pulse amplifier. Polarisations of the beams are marked by *double-headed arrows* and *concentric circles*. The 300-µm-thick Si wafers at Brewster angle are used as polarisation beam splitter and beam combiner to transmit the 2.1-µm pump pulse and reflect the signal and idler pulses. The synthesised pulses and a branch of 2.1-µm reference pulses are sent into cross-correlation frequency-resolved optical gating with a 30-µm-thick GaSe nonlinear crystal. The synthesis of a sub-cycle mid-IR pulse from coherently combining the sub-2-cycle signal and idler pulses is shown conceptually on the top of the figure

The schematic of the mid-IR sub-cycle pulse generation and characterisation is presented in Fig. 1. An octave-spanning Ti:sapphire oscillator provides the seed for a 2.1-μm OPCPA through an intrapulse DFG stage that ensures passive CEP stabilisation. The kHz, multi-mJ, CEP-stable, 2.1-µm OPCPA^36^ serves as the pump of the mid-IR OPA. A 20 µJ portion of the 2.1-µm pump is split for WLG in a 6-mm-thick BaF_2_ plate^27^ as the CEP-stable, unamplified signal for the mid-IR OPA. The CEP of the idler pulse is also passively stabilised by a DFG-like parametric process between the WLG signal and the pump pulses, regardless of the CEP stability of the pump. A 1.1-mm-thick CSP is chosen for the type-I parametric conversion for its large nonlinear coefficient, ultrabroad phase-matching bandwidth in the mid-IR, and high damage threshold pumped by 2.1-μm pulses. The pump beam size is ~6 mm in diameter and the intensity is 150 GW cm^−2^ at 800 μJ of pump energy. The output pulse from the mid-IR OPA together with an ~10 µJ, 2.1-µm pulse split from the pump that serves as the reference beam are sent into the XFROG for the temporal characterisation. More details of the setup are found in Methods section.

**Fig. 2:**
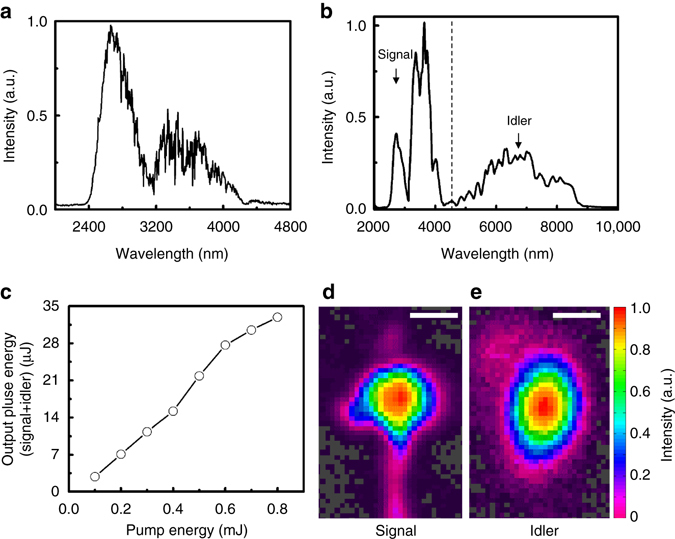
Mid-infrared optical parametric amplifier with broad bandwidth and high energy. **a** The signal spectrum from white-light generation at BaF_2_, measured after a 2400 nm long-pass filter. **b** The measured output spectrum of the mid-infrared optical parametric amplifier (mid-IR OPA). The *dotted line* separates the signal and idler spectra. **c** The output pulse energy of the mid-IR OPA vs. pump energy. The *solid line* connects the measured points to guide the eye. The far-field beam profile of the amplified signal and idler is presented in **d** and **e**, respectively. The *white bars* represent a scale of 1 mm

Figure 2a shows the mid-IR spectrum of the signal pulse that is a part of the supercontinuum from the WLG. We obtain ~50 nJ of pulse energy within the spectral window of 2.5− 4.4 µm. The spectra of the amplified signal and idler with 0.8 mJ pump energy are shown in Fig. 2b, spanning from 2.5 to 9.0 µm. The dip at ~3 µm originates from the WLG seed. It is worth noting that the long-wavelength component of the pump at 2.2–2.3 µm gives excellent phase matching in the spectral range of 4–8 µm, as compared in Supplementary Fig. 1. Therefore, the 2.2–2.3 µm wavelength component of our broadband pump is very helpful to achieve the octave-spanning parametric conversion. The 33 µJ output energy from the mid-IR OPA at 0.8 mJ pump is demonstrated as shown in Fig. 2c, of which there is a 12 µJ idler pulse spanning from 4.4 to 9.0 µm. The conversion efficiency to the synthesised pulse is ~6% considering the Fresnel reflection of the pump at the uncoated CSP crystal. While the available pump energy is higher than 2 mJ, the OPA stage has been designed at ~1 mJ of pump energy in this work because of the energy loss from metallic mirrors (13 bounces) used for the beam delivery. The shot-to-shot energy stability of the mid-IR OPA is ~2.7% rms (10,000 shots) with an oscillation of ~7% peak-to-peak over every ~10 min of period, which is attributed to the on/off operation of the air conditioner in the lab, as shown in Supplementary Fig. 9. There is no noticeable degradation of energy over several hours. The near-Gaussian signal and idler beam profiles, measured with a pyroelectric camera (PyroCam III, Spiricon) are presented in Fig. 2d, e, respectively. It should be noted that the idler spectrum can be even broader (up to 10 μm along with a stronger signal pulse), if we use a higher 2.1 μm pulse energy for WLG, as shown in Supplementary Fig. 3. However, we limit the energy to ~50 nJ within the signal bandwidth because the CEP of the signal generated by WLG is found to be less stable at higher energy.

### CEP-stable few-cycle signal and idler pulses

**Fig. 3:**
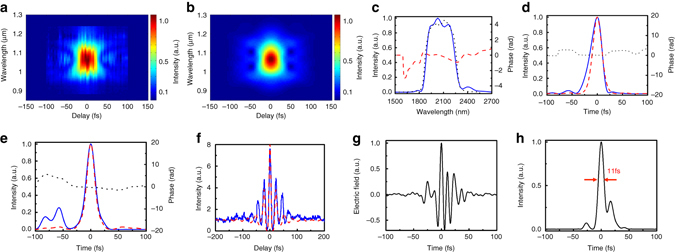
Temporal characterisation of pump, signal and idler pulses, and calculated temporal profile of the synthesised pulse. **a**–**d** The temporal characterisation of the 2.1-µm pump pulse using the second-harmonic generation frequency-resolved optical gating (SHG FROG). The measured (**a**) and retrieved (**b**) FROG traces. **c** The retrieved spectrum (*blue solid*) and phase (*red dash*) in the FROG measurement. The separately measured spectrum is shown by the *black dotted line* that agrees well with the retrieved spectrum in FROG. **d** The retrieved (*blue solid*) and the calculated transform-limited (*red dash*) intensity profiles. The *black dotted curve* is the retrieved phase. The pulse duration of 26 fs FWHM of the pump is measured. **e**, **f** The temporal characterisation of the amplified signal and idler pulses using the SHG FROG and interferometric autocorrelator, respectively. **e** The retrieved (*blue solid*) and the calculated transform-limited (*red dashed line*) intensity profiles of the amplified signal pulses. The *black dotted curve* is the retrieved phase. A pulse duration of 20 fs in FWHM of the amplified signal is obtained. See Supplementary Fig. 7 for the measured and retrieved SHG FROG traces. **f** The measured autocorrelation trace of the idler pulse (*blue solid*) and the calculated autocorrelation trace from the measured spectrum assuming the pulse is transform limited (*red dashed line*). A pulse duration of 31 fs FWHM of the idler is obtained, assuming a Gaussian temporal profile. **g** The calculated electric field of the synthesised mid-IR pulse using the measured spectra of the signal and idler pulses. It is assumed that both the signal and idler pulses are transform limited, the idler leads the signal by 5 fs, and the CEP is zero for both pulses. **h** The calculated intensity profile. The calculated duration of the synthesised pulse is 11 fs in FWHM corresponding to 0.8 cycle at 4.1 μm

The temporal profile of the 2.1-µm pulses from the OPCPA, which serves both as the pump of the mid-IR OPA and as reference of the XFROG, is characterised using a second-harmonic generation (SHG) FROG apparatus. As shown in Figs. 3a–d, the pump pulse whose spectrum spans from 1.8 to 2.3 µm is within 5% of its transform limit, with a pulse width of 26 fs in FWHM. The signal pulse from WLG has a slight self-compression in BaF_2_ that has small anomalous dispersion at 2.1 μm^27^. The pulse duration of the amplified signal is measured to be ~20 fs using the SHG FROG, as shown in Fig. 3e.

Before we explore the coherent pulse synthesis, we investigate the temporal profile of the idler pulse which already contains an octave-spanning spectral content. The amplified idler pulse is independently characterised using a home-built mid-IR second-order interferometric autocorrelator. An uncoated 1-mm-thick ZnSe plate is employed as a beam splitter, and a 4.5-µm long-pass filter is used to isolate the idler pulse from the signal and any residual pump. The anomalous dispersion from the ZnSe plate is well compensated by the normal dispersion from the germanium substrate of the long-pass filter, however there is ~8000 fs^3^ of uncompensated third-order dispersion in this configuration. As shown in Fig. 3f, the measured interferometric autocorrelation trace is plotted together with a transform-limited trace calculated using the measured spectrum. The good agreement between the three central lobes of the pulses manifests that the idler is nearly transform limited with a pulse width <1.5 optical cycles, centred at 6.4 µm. Approximately 31 fs pulse width is deconvoluted assuming the idler pulse is in the Gaussian profile. The unsuppressed pedestal from the measured pulse accounts for the uncompensated third-order dispersion. This measurement shows that the octave-spanning, ~6.4-μm, sub-1.5-cycle idler pulse, which is CEP stable as will be discussed again, is already usable for phase-sensitive strong-field experiments in solids and nano-structures as a stand-alone mid-IR source.

In the collinear type-I OPA, the signal and idler pulses have the same polarisation and can be synthesised automatically if the temporal walk-off between them is much smaller than an optical cycle and the CEP of both pulses is stable. The temporal walk-off within the 1.1-mm-thick CSP crystal is as small as ~5 fs (less than a quarter cycle of the idler wavelength, ~6.4 μm). The electric field of the synthesised pulse is calculated as shown in Fig. 3g, using the measured spectra and the calculated 5 fs temporal walk-off. Here we assume both signal and idler pulses are transform limited, and the CEP of both pulses is zero. The 11 fs in FWHM of the synthesised pulse is then obtained as shown in Fig. 3h, corresponding to 0.8 optical cycles centred at 4.1 µm. This clearly reveals the feasibility of sub-cycle pulse synthesis. An independent simulation also supports this calculation, as shown in Supplementary Fig. 2, along with the energy scaling to mJ level from the second stage OPA. It should be noted that in the case of multi-stage OPA, the pulse synthesis always occurs in the final stage. The temporal walk-off in the first or intermediate stage is ignored because we use the idler only from the final stage for the pulse synthesis.

**Fig. 4:**
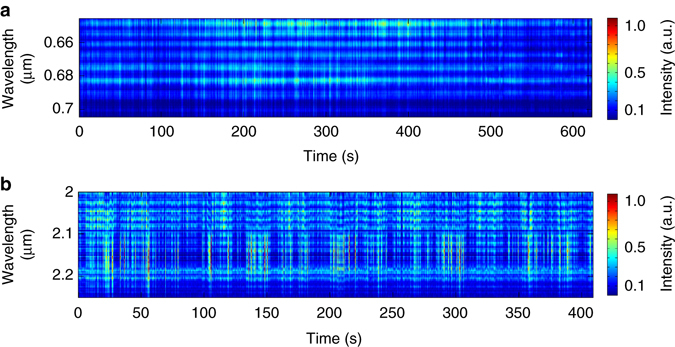
Characterisation of shot-to-shot carrier-envelope phase stability. **a** The measured self-referencing *f-*3*f* spectral interferometry (SI) of the 2.1-µm pump pulse over 10 min. Shot-to-shot carrier-envelope phase (CEP) jitter (220 mrad) is measured over 10 min. **b** The measured cross-referencing *f*-3*f* SI of the idler pulse, between the polarisation-rotated ~2.1-µm pump and the third harmonic of the idler at ~6.4 µm over 6 min. Shot-to-shot CEP jitter (270 mrad) of the idler pulse over 6 min is calculated with the measured phase jitters of pump and the cross-referencing SI

The stable CEP of individual pulses as well as the relative phase is crucial for the coherent pulse synthesis in the single-cycle limit. The CEP stability of the 2.1-µm pump is measured using the self-referencing *f*-3*f* spectral interferometry (SI) of the white light that also serves as the signal of the mid-IR OPA. In other words, this *f*-3*f* measurement directly provides the CEP stability of the signal pulses. The third harmonic (TH) of the ~2 μm portion of the signal and the ~680 nm portion of the white light are spectrally interfered, as previously demonstrated^34, 37^. Figure 4a shows the stable *f*-3*f* fringes over 10 min with a three-shot average, and the shot-to-shot CEP jitter of the signal is measured as ~220 mrad rms. Similarly, the CEP stability of the idler pulse is measured using the cross-referencing *f*-3*f* SI, which is obtained by interfering the polarisation-rotated ~2.1-µm pump and the TH of the idler at ~6.4 μm. The details are shown in the Methods section and in Supplementary Fig. 5. The fairly stable single-shot interference fringes were observed over 6 min, as shown in Fig. 4b, owing to the passively CEP-stable nature of the white-light-seeded OPA. These two SI measurements indicate that the pump, signal and idler all have shot-to-shot stable CEP. The measured phase jitter in Fig. 4b is the square root of the sum of the squares of the pump and the idler CEP jitters, i.e. ${{\it{\sigma }}_{\rm Measured}} = \sqrt {{\it{\sigma }}_{\rm Pump}^2 + {\it{\sigma }}_{\rm Idler}^2}$ because they are not correlated. The shot-to-shot CEP jitter of the idler pulse is then calculated as 270 mrad rms over 6 min. It is worth mentioning that the absolute CEP value of the synthesised pulse can be controlled using a low-dispersion wedge pair. For example, we can use a 1.2-mm-thick wedge pair made of caesium iodide that has very flat dispersion from 2 to 10 µm to change the CEP by 2*π* while the sub-cycle duration is maintained (Supplementary Fig. 10). Due to the ultrabroad spectral bandwidth, it is technically challenging to shift the CEP much more than 2*π* without distorting the pulse shape. The individual CEP of the signal and idler pulses can simultaneously be accessed via the CEP adjustment of the pump pulse before and after the WLG stage, respectively, as discussed in Supplementary Note 1. While the passively stabilised CEP of both signal and idler pulses is expected to be maintained beyond ~10 min of our measurement, the active compensation of potential CEP drift due to the thermal fluctuations in the lab, as evidenced by the energy measurement in Supplementary Fig. 9, using thin wedges could further improve the long-term CEP stability. The CEP of the synthesised waveform could be directly measured using a recently demonstrated electro-optic sampling method^38^.

### Sub-cycle pulse generation and characterisation

**Fig. 5:**
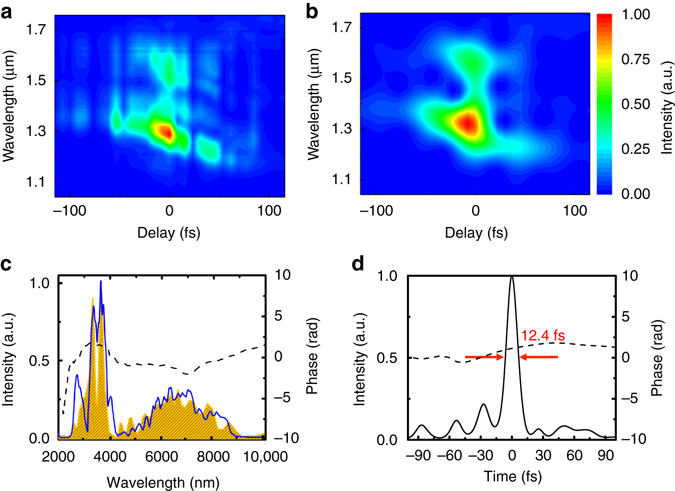
Temporal characterisation of the synthesised mid-infrared pulse. The measured (**a**) and the retrieved (**b**) cross-correlation frequency-resolved optical gating (XFROG) traces. The FROG error is 1.8%. The retrieved spectral (**c**) and temporal (**d**) intensity profiles of the synthesised pulse. The *dotted curves* are the retrieved phase. Pulse width (12.4 fs) in full-width at half maximum is measured with a centre wavelength at 4.2 µm. It corresponds to 0.88 optical cycle

The temporal profile of the synthesised pulse is characterised with XFROG. The time delay between the signal and pump pulses is finely tuned using a piezo stage within a total delay of ~30 fs, such that the shortest duration is obtained while the amplified energy is maintained at maximum. The measured and retrieved XFROG traces are shown in Fig. 5a and b, respectively, with 1.8% FROG error. The retrieved spectrum shown in Fig. 5c agrees well with the measured spectrum in Fig. 2b. There is minor discrepancy with the spectral edge at 2.5−3 μm of the retrieved spectrum due to the phase matching edge of the GaSe crystal in the sum-frequency generation, as shown in Supplementary Fig. 6. The synthesised pulse has a near-transform-limited main peak and rippling wings as shown in Fig. 5d, attributed to the interference of the signal and idler pulses. The synthesised pulse duration is measured as ~12.4 fs in FWHM centred at ~4.2 µm, corresponding to 0.88 optical cycle, which is within 10% of its transform limit as shown in Fig. 3h. With the energy portion into the main pulse of ~70% the peak power reaches ~1.9 GW. We obtain a similarly broad spectrum and high energy from an OPA with a 0.5-mm-thick ZGP crystal, as shown in Supplementary Fig. 4, which is also similar to an earlier demonstration of ultra-broadband mid-IR OPA with a 12-mm-thick ZGP crystal^39^. However, due to a relatively large temporal walk-off of ~11 fs between the signal and idler with our ZGP crystal, it is less favourable than the CSP crystal for the sub-cycle pulse synthesis.

### HHG in solids

We have used the synthesised mid-IR laser pulses to drive HHG in silicon (Si) to show the potential of this source for sub-cycle electron control in solids. A free-standing 200-nm-thick Si (100) sample and a 500-nm-thick Si (100) sample on a 0.5-mm-thick sapphire substrate are used for generating high harmonics. The OPA signal and idler beams with a Gaussian beam diameter of ~5.5 mm are focused using an *f* = 25.4 mm gold-coated off-axis parabolic mirror. The HHG signal is collected using an ultraviolet (UV)-enhanced aluminum-coated off-axis parabolic mirror and spectrally resolved using a visible-to-UV monochromator with an intensified charge-coupled device. It should be noted that due to the large difference in the centre wavelength between the signal (~3.2 μm) and the idler (~6.4 μm) beams, the focused beam size of the two beams with the same focal length differs by a factor of ~2. Along with the two times higher energy of the signal beam than the idler beam, the HHG is dominated by the signal pulse at the focus (*z* = 0 mm). To observe the clear contribution of the idler pulse, we have acquired most high-harmonic spectra at *z* = 0.5−1.0 mm after the focus where the spot sizes of the signal and idler beams are comparable. The beam size in 1/*e* ^2^ radius at *z* = 0.5 mm is estimated as ~57 and ~64 μm for the signal and idler, respectively. The estimated intensity of the ~20 μJ synthesised pulse with 60 μm of beam waist is ~9×10^12^ W cm^−2^, corresponding to the electric field strength of 0.8 V Å^−1^. More details on the HHG setup are found in the Methods section.

**Fig. 6:**
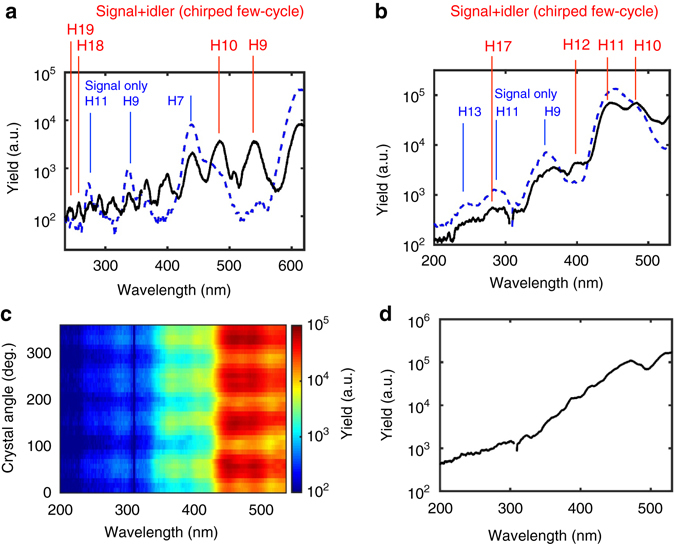
Mid-infrared-driven high-harmonic generation in silicon samples. **a** Harmonic spectra generated in a 200-nm-thick free-standing silicon (Si) sample. The *solid black line* represents the spectrum generated by a chirped, ~43 fs, synthesised pulse, where the odd and even harmonics up to 19th order are observed. The *blue dotted line* represents the spectrum generated by only the signal pulse, where only odd harmonics up to 11th order are observed. **b**–**d** Harmonic spectra generated in a 500-nm-thick Si sample on a 0.5-mm-thick sapphire substrate. **b** The *solid black line* represents the spectrum generated by a chirped, ~43 fs, synthesised pulse, where the odd and even harmonics up to 17th order are observed. The *blue dotted line* represents the spectrum generated by only the signal pulse, where only odd harmonics up to 13th order are observed. **c** The angle dependence of the harmonic spectra about the Si axis <001> relative to the laser polarisation. The four-fold symmetry confirms that the harmonic spectra originate from HHG in Si. The high-harmonic generation (HHG) yield is maximised when laser polarisation is along <011>. **d** The near-continuous harmonic spectrum generated by the synthesised sub-cycle pulse without chirping. The harmonic comb structure is almost washed out due to the isolated emission from HHG

In the experiment we observed a continuum-like harmonic spectrum using synthesised sub-cycle drive pulses, which will be discussed at the end. To verify that the spectrum originates from HHG in silicon, we have generated harmonics with a clear comb structure using few-cycle pulses and demonstrated four-fold symmetry of the harmonic yields, which reflects the crystal symmetry of Si. This is a feature of HHG rather than incoherent light emission such as fluorescence. A ~16 μJ, few-cycle pulse is obtained by positively chirping the synthesised pulse using a 0.5-mm-thick Si filter (IPA3000, EOC, Inc.) with ~80% transmission over 3−11 μm. The positive dispersion of the Si filter moderately broadens the pulse duration to ~43 fs in FWHM of the double peaks, which is long enough to generate discrete harmonics. The measured OPA spectrum with the filter and the calculated temporal profile are shown in Supplementary Fig. 8. The intensity of the chirped pulse is estimated to be ~2×10^12^ W cm^−2^ corresponding to an electric field strength of 0.4 V Å^−1^. Figure 6a and b show the harmonic spectra generated using the ~43 fs chirped pulses in the 200-nm-thick and 500-nm-thick Si samples, respectively. Both odd and even harmonics of ~4.6 μm (*black solid lines*) are observed up to ~19th harmonic (~244 nm) because the two-colour driving field unbalances the electron trajectories and breaks the symmetry^13^. For comparison, the odd harmonics of ~3.2 μm from signal-only-driven HHG is also plotted as the *blue dotted line* of Figs. 6a and b, which are obtained at the beam focus (*z* = 0 mm) where the signal pulse dominates HHG over the idler pulse due to the smaller beam size (half) and higher pulse energy (twice). It is noted that the spectral comb structure is more clearly observed in Fig. 6a, which is potentially due to the effect from the sample thickness or the sapphire substrate and requires more investigation. The spectral intensity as a function of the crystal orientation, driven by the ~43 fs chirped pulse with the 500-nm-thick Si sample, in Fig. 6c clearly shows the four-fold symmetry of Si when the sample is rotated around the <100> crystal axis, confirming that the emission spectrum originates from HHG in Si. Finally, Fig. 6d shows the harmonic spectrum driven by the synthesised sub-cycle pulse without the 3−11 μm Si filter. The harmonic comb structure is almost washed out and a near-continuous spectrum is observed due to the sub-cycle nature of the driver pulse that isolates the harmonic emission. Further investigations on the CEP dependence along with the pump-probe analysis will enable the observation and control of isolated sub-cycle electron dynamics.

## Discussion

Multi-mJ sub-cycle or single-cycle mid-IR sources are desired for strong-field interactions in gaseous media, such as isolated zeptosecond HHG in the keV. The scaling of energy and peak power of the demonstrated mid-IR single-cycle synthesiser is relatively straightforward by adding more OPA stages with higher pump energy. The availability of Joule-level picosecond Yb-doped lasers^40^ ensures the energy scalability of our 2.1 μm OPCPA to multi-ten mJ level, which can eventually increase the synthesised mid-IR pulse energy to multi-mJ. It is worth mentioning that the mid-IR crystals like CSP and ZnGeP_2_ (ZGP) have damage thresholds >200 GW cm^−2^ pumped by femtosecond ~2-µm pulses. The availability of >20 mm × 20 mm crystal aperture^41^ allows the employment of a ~2-µm, ~40-fs pump with >20-mJ pulse energy to achieve the mJ sub-cycle mid-IR pulse amplification, as discussed in Supplementary Fig. 2. Recently, there have been mid-IR OPCPA demonstrations with sub-mJ energy and 4−8 cycle pulse width, pumped by high energy ~2 μm picosecond Ho-doped lasers.^42, 43^ Our approach can be combined with the advanced ~2–3 μm pump laser technologies^42–44^ to realise a relatively compact multi-mJ near-single-cycle mid-IR laser source if the sub-50 fs duration can be accessed by these lasers via external pulse compression techniques^29^. On the other hand, our passive synthesis scheme can be widely adopted for generating sub-1.5-cycle, sub-mJ mid-IR pulses if existing ~2 μm OPA systems pumped by conventional Ti:sapphire laser amplifiers are used for pumping CSP and ZGP crystals.

In conclusion, we demonstrated a high-energy, CEP-stable, sub-cycle, mid-IR pulse synthesiser based on an OPA covering the bandwidth from 2.5 to 9.0 µm and drove HHG in thin Si samples to show the isolated sub-cycle strong-field interactions in solids. The synthesised pulse width was measured as ~12.4 fs, corresponding to 0.88 optical cycle at ~4.2 μm. The stable CEP was ensured with the passively CEP-stable pump and the WLG-seeded OPA scheme. CEP jitters (220 and 270 mrad rms) were measured for the signal and idler pulses, respectively, over >6 min. A synthesised pulse with 33 µJ energy and ~1.9 GW peak power was obtained. HHG up to ~19th order was demonstrated in thin Si samples. The continuous harmonic spectrum confirmed that the generated sub-cycle mid-IR pulses allow for isolated harmonic emission. Further investigations in temporal domain along with CEP dependence are required for the detailed studies of strong-field electron dynamics in solids. We note that the energy scaling of this mid-IR source is relatively straightforward by adding OPA stages. Advanced pump laser technologies can potentially push the mid-IR sub-cycle source towards TW peak powers to drive strong-field interactions in gaseous media.

## Methods

### Mid-IR OPA construction

An octave-spanning Ti:sapphire oscillator (650–1100 nm) generates the 2.1-µm signal seed through intrapulse DFG in a MgO:PPLN. The Ti:sapphire oscillator also seeds both picosecond 1047 nm Nd:YLF pump laser and 1030 nm cryo-cooled Yb:YAG pump laser. They provide ~1.5 and ~45 mJ pump energy for the 2.1-μm OPCPA, respectively. The amplified 2.1-μm pulses from the three-stage OPCPA are passively CEP stabilised with 3.5 mJ of maximum pulse energy and 26 fs pulse width. The 0.8 mJ, 2.1-µm pulses are used as the pump of the mid-IR OPA. A 20 µJ portion of the 2.1-µm pump is split for WLG in a 6-mm-thick BaF_2_ plate as the signal of the mid-IR OPA. The polarisation is rotated by 90^o^ using a CaF_2_ half-wave plate to fulfill the type-I phase matching. A 1.1-mm-thick CSP from BAE Systems with *θ = *47^o^ is chosen for the type-I parametric conversion for its large nonlinear coefficient, broad phase-matching bandwidth, and high damage threshold pumped by 2.1 μm pulses. As there is a lack of mid-IR broadband beam combiners and beam splitters, 300-µm thick Si plates are oriented at the Brewster angle of the 2.1 μm pump serving as the beam combiner and beam splitter. With a large refractive index (i.e. *n*~3.44), Si has a large Brewster angle that is ~74° at 2.1 µm. This requires a ~74° incident angle of the signal and idler beams with orthogonal polarisation to the 2.1-µm pump in the collinear OPA scheme, which gives ~70% reflection of the signal and idler pulses. The energy loss from the Si beam splitter is taken into account in Fig. 2c.

### Spectral characterisation

The output spectra of the mid-IR OPA are recorded by a scanning-grating monochromator (Horiba) with a liquid-nitrogen-cooled MCT detector. Long-pass filters with cutting wavelengths at 2400, 3600, 4500 and 7300 nm are used to analyse the two-octave-spanning spectrum.

### SHG FROG

The temporal profile of the 2.1-μm pump and the amplified signal pulses is characterised using a SHG FROG apparatus. A 100-µm thick β-barium borate and a 140-µm thick AgGaS_2_ is used for the SHG of the 2.1-μm pump and the amplified signal, respectively.

### XFROG

A beam with 10 µJ energy is split from the 2.1-μm pump as the reference of the XFROG. The synthesised pulse and the reference pulse are focused on a 30-µm-thick type-I GaSe crystal. The dispersion of the crystal is as low as 2.5 fs^2^ at 4.2 μm. The generated sum-frequency signal is coupled into a spectrometer with InGaAs detectors (NIRQuest512, Ocean Optics). The spectral resolution of 6.7 nm limits the overall resolution of the measured XFROG trace. An iris aperture and a polariser are used to block the leakage of the fundamental pulses and thus increase the signal-to-noise ratio. The delay scan is done at the synthesised pulse arm. For the XFROG measurement of an optimally synthesised pulse, the time delay between the signal and pump pulses is finely tuned using a piezo stage within a total delay of ~30 fs, such that the shortest duration is obtained while the amplified energy is maintained at maximum. The grid size of XFROG retrieval is 128.

### The cross-referencing ***f***-3***f*** nonlinear spectral interferometry

We use the CEP-stable 2.1-µm pump beam as the CEP reference for the idler. The optical schematic is shown in Supplementary Fig. 5a. By slightly rotating the CSP crystal and thereby inducing very small birefringence to the 2.1-µm pump, we can generate the weak, vertically polarised component of the residual pump in Fig. 1 (~5 μJ of energy out of 0.8 mJ) while keeping the same performance of the mid-IR OPA. This polarisation-rotated residual pump is also reflected by the Si beam splitter and collinear with the idler pulse that spans from 4.4 to 9.0 µm. Since the spectrum of the polarisation-rotated residual pump is found to be narrower than the original pump spectrum, it is first focused to a 1-mm-thick ZGP crystal for the moderate spectral broadening through self-phase modulation. After that the collinear pulses, including the broadened residual pump and the idler, are focused to another 0.5-mm-thick ZGP to generate the TH of the idler in the wavelength of 1.7–2.4 µm, with the optimised phase-matching angle of the ZGP crystal. The spectrally overlapped TH of the idler and the broadened residual pump with a fixed time delay are coupled into a spectrometer with InGaAs detectors (NIRQuest512, Ocean Optics) for the cross-referencing SI measurement. The measured spectra of the broadened polarisation-rotated residual pump and the TH of the idler are shown in Supplementary Fig. 5b. The single-shot interference measurements are taken with an integration time of 1 ms, as presented in Supplementary Fig. 5c.

### HHG setup

The laser beam is focused using an *f* = 25.4 mm off-axis parabolic mirror with gold coating and then collimated using an *f* = 50.8 mm off-axis parabolic mirror with UV-enhanced Al coating that can reflect the high harmonics up to the UV range. The mid-IR and harmonic beams are focused into an UV-visible monochromator (SP-300i, Acton Research Corporation) with an intensified charge-coupled device camera (PI-MAX, Princeton Instruments). Low-order harmonics in the NIR region are not measured because the cutoff region is of main interest. A 200-nm-thick free-standing Si (100) sample and a 500-nm-thick Si (100) sample on a 0.5-mm-thick sapphire substrate are used as the medium of HHG. Each solid sample is mounted on a rotation stage and a translation stage for optimisation of the HHG signal. The laser beam is focused into the sample with the normal angle of incidence and the vertical polarisation. The crystal orientation relative to the laser polarisation is rotated using the rotation stage, whereas the position of the sample relative to the beam focus along the propagation direction is adjusted using the linear stage. While the focused spot sizes of signal and idler beams are estimated as ~13 and ~26 μm in Gaussian waist, respectively, with *M* ^2^ value of ~1.7, the sample position is optimised for the HHG experiment as explained in section HHG in solids.

### Data availability

The data that support the findings of this study are available from the corresponding author upon request.

## Electronic supplementary material

Supplementary Information
