# Supplementary material for: High-energy mid-infrared sub-cycle pulse synthesis from a parametric amplifier
Source: Nat Commun. 2017 Jul 26;8:141. doi: 10.1038/s41467-017-00193-4 (PMC5529551; doi:10.1038/s41467-017-00193-4)
Supplement: Supplementary file 1 — Supplementary Information [file 41467_2017_193_MOESM1_ESM.pdf]

File name: Supplementary Information
Description: Supplementary Figures, Supplementary Note and Supplementary References

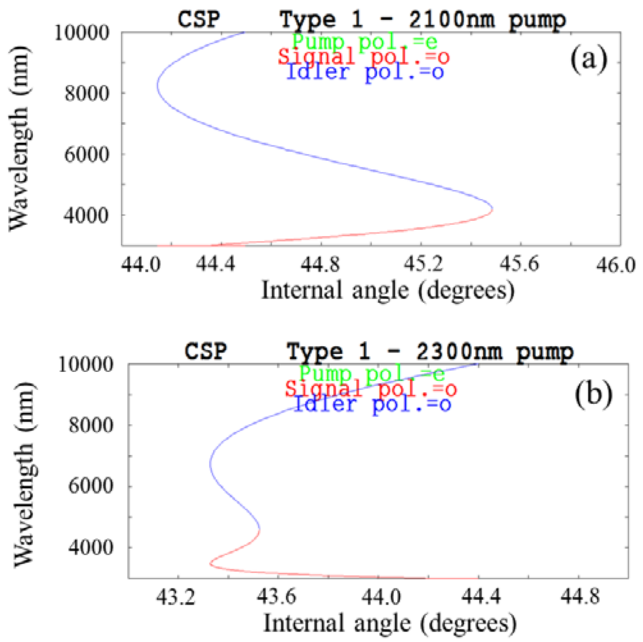

Supplementary Figure 1: Phase-matching condition of the mid-infrared optical parametric amplifier with $CdSiP_2$ crystal pumped at different frequency component of the broadband 2.1-μm laser. The phase-matching curves from SNLO of the collinear type-I mid-infrared (mid-IR) optical parametric amplifier (OPA) with $CdSiP_2$ (CSP) crystal (θ=47°) pumped by pulses at (a) 2.1 μm and (b) 2.3 μm. (SNLO nonlinear optics code available from A. V. Smith, AS-Photonics, Albuquerque, NM, USA.)

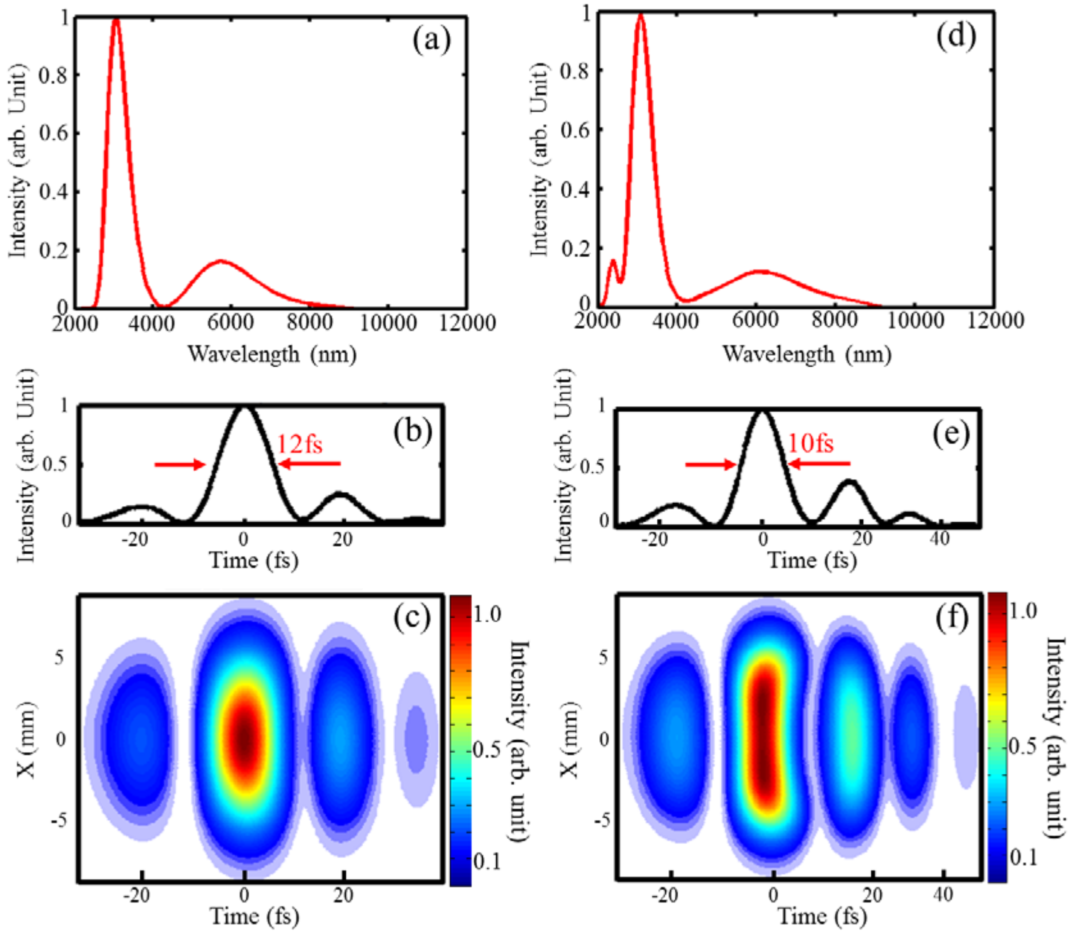

Supplementary Figure 2: Multi-stage mid-IR OPA simulations with CSP crystals. OPA simulations have been carried out with a 2+1 dimensional nonlinear pulse propagation analyzer[1]. It performs the calculation in the dimensions of 1) transverse direction which defines the phase-matching angle with the optical axis of the crystal, 2) propagation direction, and 3) time domain. The simulation includes all possible second-order nonlinear processes, phase matching, diffraction, and walk-off for the pulses in the two orthogonal polarizations. The third-order processes like self-phase modulation and self-focusing are not considered. 2.1 μm, 26 fs transform-limited pump pulses and 3 μm, 20 fs transform-limited signal pulses are used in the simulation. The pump and input signal energy is 550 μJ and 50 nJ, respectively, for the first OPA stage, while the pump and input signal energy is 20 mJ and 20 μJ, respectively, for the second stage. The output energy of the synthesized pulse from the second OPA stage is 1 mJ. The thermal effect by the absorption in the second-stage CSP crystal is negligible. The absorption coefficient of CSP at 2, 4.5, and 7–9 μm is 0.05, 0.02, and 0.6–2 cm^{-1}, respectively[2]. The absorbed energy in the 1.1-mm-thick CSP with 20 mJ, 2.1 μm pump is only 10 μJ and that of signal and idler pulses is also as low as ~2 μJ (out of ~0.66 mJ) and ~15 μJ (out of ~0.33 mJ at 7-9 μm), respectively. With the beam size of 20 mm diameter and this amount absorption (at 1 kHz repetition rate) the temperature increase inside the crystal is negligible ($<10^{-2}$ K). The optical spectrum (a) and spatio-temporal profile (b) and (c) of the pulse from the first OPA stage. The simulated spectrum agrees relatively well with the measured spectrum shown in Fig. 2(b). The simulated pulse width in full width at half maximum (FWHM) is 12 fs which agrees well with the cross-correlation frequency-resolved optical gating (XFROG) measurement. The optical spectrum (d) and the temporal profile (e) of the second stage confirm that we can maintain the sub-cycle mid-IR waveform with the same spectral coverage. The output energy of the synthesized mid-IR waveform in the first and the second stage is 30 μJ and 1.0 mJ, respectively.

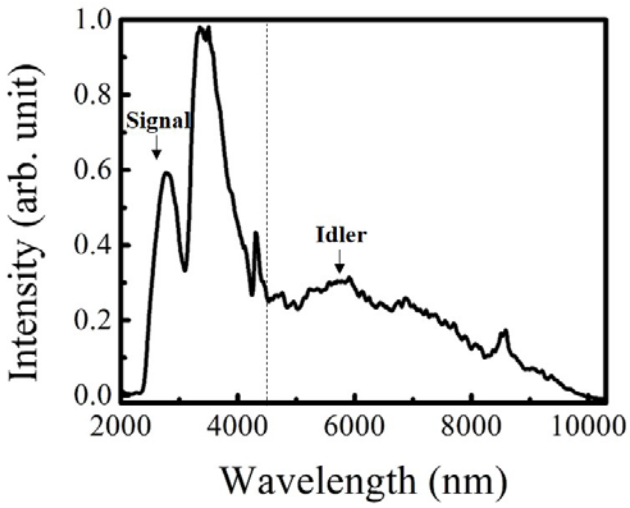

Supplementary Figure 3: Output spectrum of the mid-IR OPA with 100 nJ seed signal. A 1.1-mm-thick CSP crystal is used as the mid-IR nonlinear crystal.

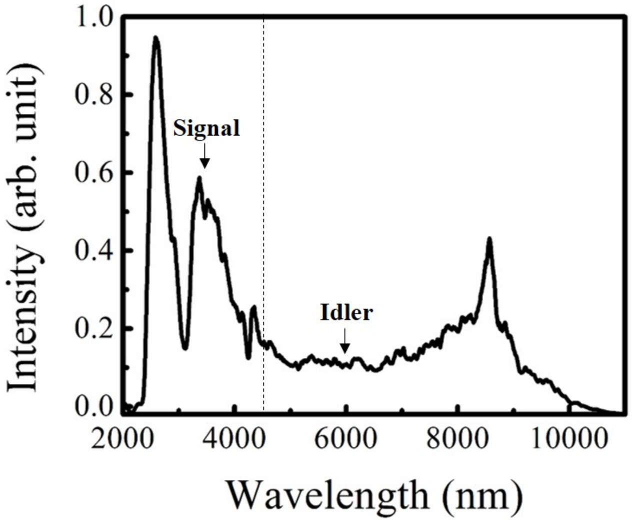

Supplementary Figure 4: Output spectrum of the mid-IR OPA with 100 nJ seed signal. A 0.5-mm-thick $ZnGeP_2$ (ZGP) crystal is used as the mid-IR nonlinear crystal.

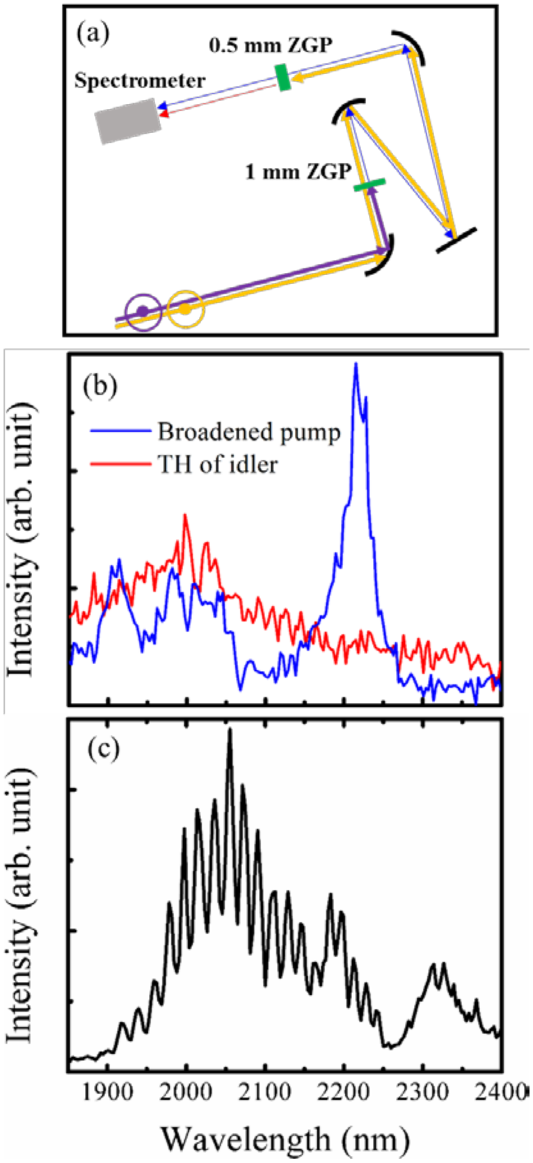

Supplementary Figure 5: Cross-referencing *f*-3*f* spectral interferometry for characterizing shot-to-shot carrier-envelope phase stability of idler pulses. (a) The setup schematic of the cross-referencing spectral interferometery (SI) measurement (colour online). The 2.1-μm polarization-rotated residual pump pulse (purple color), which is found to have narrower spectrum than the original pump spectrum, is focused to a 1-mm-thick ZGP crystal for the moderate spectral broadening through self-phase modulation (blue color), together with the collinear idler pulse (yellow color) spanning from 4.4 to 9 μm. The collinear pulses are then focused to another 0.5-mm-thick ZGP to generate the third-harmonic (TH) of the idler in the wavelength of 1.7 to 2.4 μm (red color), with the optimized phase-matching angle of the ZGP crystal. The spectrally overlapped TH of the idler and the broadened pump pulses are coupled into a spectrometer with InGaAs detectors (Ocean Optics, Inc.) for the SI measurement. The polarization of the 2.1-μm polarization-rotated residual pump and the idler pulses is marked by concentric circles. (b) The spectra of the spectrally broadened, 2.1-μm residual pump pulse (blue) and the TH of the idler pulse (red). (c) The interference spectrum of the broadened polarization-rotated pump and the TH of the idler pulses with 1 ms of integration time for the single-shot measurement.

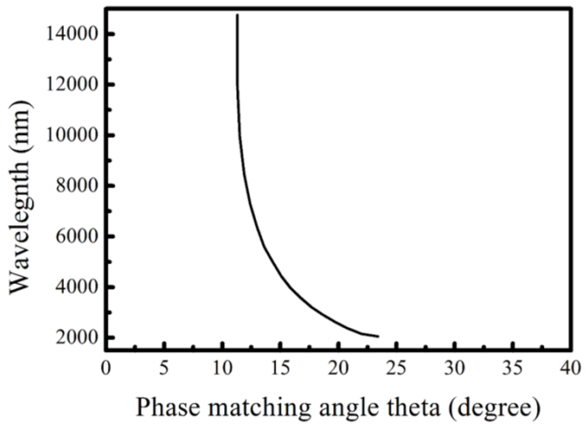

Supplementary Figure 6: Phase matching curve of GaSe in the type-I sum-frequency generation. 2050 nm is chosen as the reference frequency as in the XFROG measurement. (SNLO nonlinear optics code available from A. V. Smith, AS-Photonics, Albuquerque, NM.)

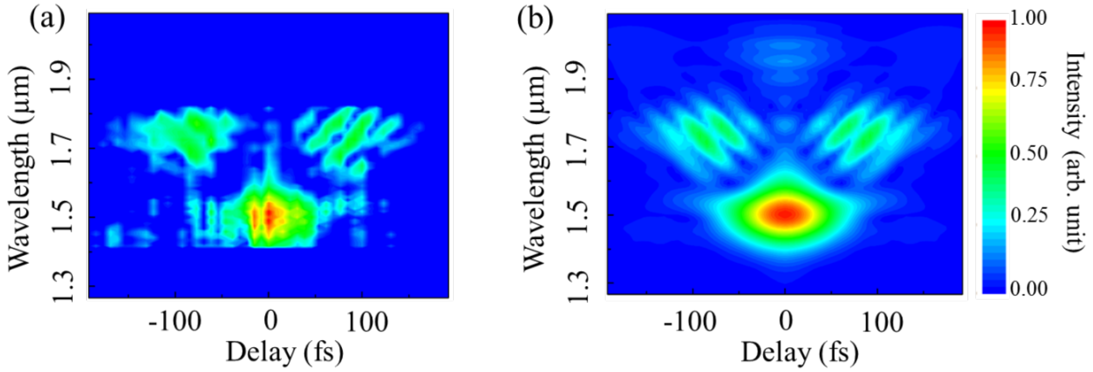

Supplementary Figure 7: Temporal characterization of amplified signal pulses. The measured (a) and retrieved (b) second-harmonic generation (SHG) FROG traces of the amplified signal pulses. 140-μm thick $AgGaS_2$ crystal is used as the SHG crystal in the FROG measurement.

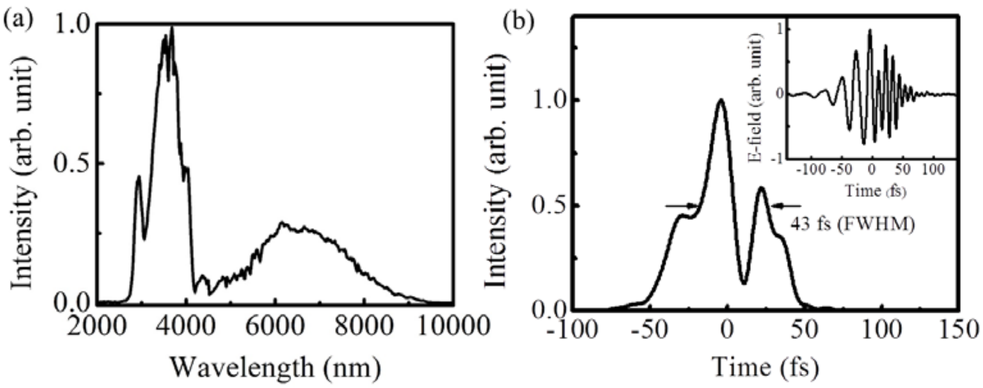

Supplementary Figure 8: Chirped, few-cycle mid-IR pulse dispersed by a 0.5-mm-thick silicon filter with a high transmission (~80%) at 3-11 μm. To generate discrete harmonics, we intentionally chirped the sub-cycle drive pulses to few-cycle duration using a 0.5-mm-thick silicon (Si) filter (IPA3000, EOC, Inc.) which has positive (normal) dispersion and good transmission (~80%) in the whole mid-wave infrared range. (a) Measured optical spectrum of signal and idler pulses and (b) calculated temporal intensity profile with the electric field (inset). The pulse duration is ~43 fs in FWHM of the double peaks with positive chirp.

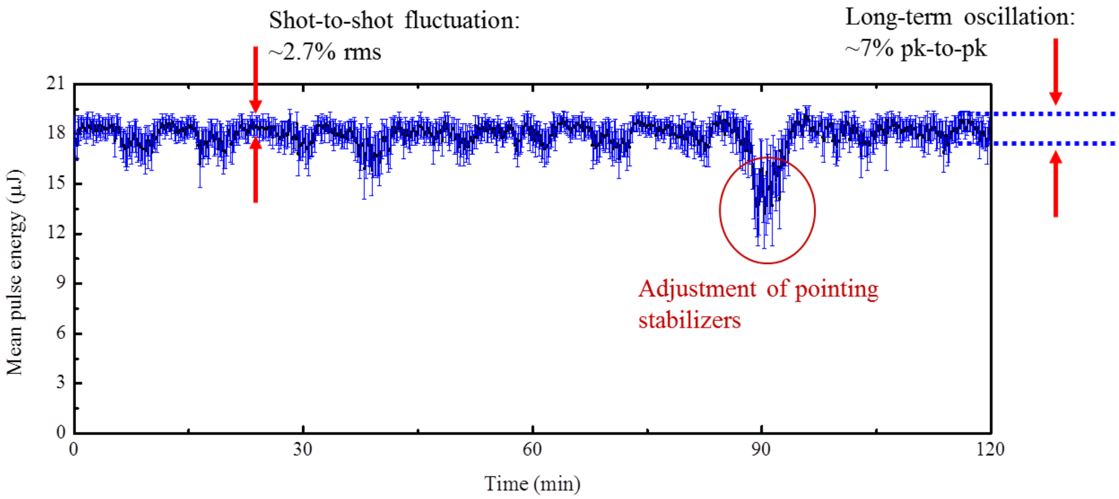

Supplementary Figure 9: Long-term energy stability measurement of synthesized mid-IR pulses. Energy stability of signal and idler pulses is recorded over 2 hours using a pyroelectric detector (Molectron, Coherent Inc.). The pump energy is ~0.65 mJ and the 3-11 μm filter (~80% transmission) is used for blocking background optical noises. The shot-to-shot energy fluctuation is measured as ~2.7% rms over 10,000 shots, while the intermediate-term oscillation of the mean pulse energy is measured as ~7% peak-to-peak over ~10 minutes of period which corresponds to the on/off operation of the air conditioner in the laboratory. However, there is no long-term drift of the pulse energy observed over several hours. The significant drop of the pulse energy at ~90 minutes of time is due to the manual adjustment of the beam pointing stabilizers in the cryogenic Yb:YAG pump laser and the 2.1 μm optical parametric chirped pulse amplifier when they go out of the range, which occasionally happens.

Supplementary Note 1: CEP control of the synthesized pulse

The absolute carrier-envelope phase (CEP) of the synthesized pulse can be shifted using a low-dispersive wedge pair. For example, we can use a 1.2-mm-thick wedge pair made of caesium iodide (CsI) which has very flat dispersion from 2 to 10 μm to change the CEP by 2π while the sub-cycle duration is maintained, as shown in Supplementary Fig. 10 below. However, due to the ultrabroad spectral bandwidth, it is challenging to shift the CEP much more than 2π without distorting the pulse shape with sub-cycle duration.

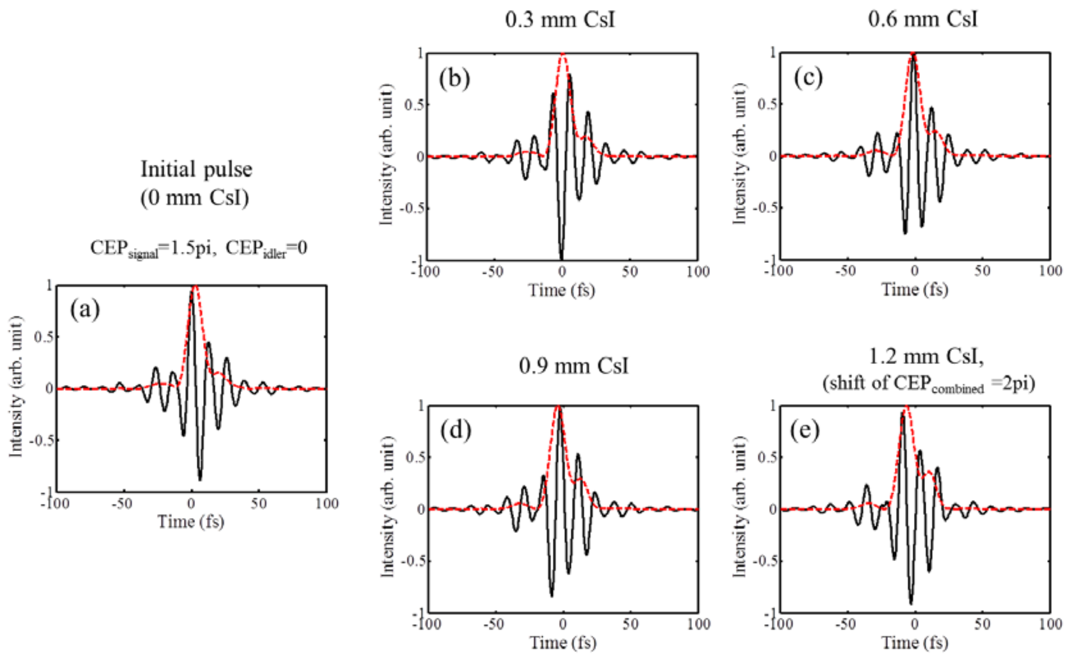

Supplementary Figure 10: Calculated CEP change of the synthesized pulse with regard to the thickness of CsI. The CEP of signal and idler pulses before a CsI window is preset to be 1.5π and 0π, respectively, as shown in (a). The CEP of the combined pulse can be shifted up to 2π using the 1.2-mm-thick CsI window (e) while the sub-cycle pulse duration is maintained. However, the group-delay dispersion (GDD) and third-order dispersion (TOD) eventually broadens and distorts the pulse shape if the excessive thickness is used for CEP shift.

Besides the CEP shift of the synthesized pulse, the individual CEP of the signal and idler pulses can simultaneously be accessed via the CEP adjustment of the pump pulse before and after the white light generation (WLG) stage, respectively. We discuss how we can access the individual CEP as below.

Since the signal pulse has a constant relative phase to the pump pulse in the WLG process, the signal CEP is changed accordingly with the CEP shift of the pump pulse. It is relatively straightforward to change the CEP of the CEP-stable 26 fs, 2.1 μm pump pulse before the WLG stage using the techniques basically the same as what has been known for near-IR lasers, *i.e.*, CEP-stable Ti:sapphire laser amplifiers. Here, we suggest two specific methods: 1) the use of a thin wedge pair made of low-dispersion BaF_2 which has zero dispersion at ~1.9 μm and 2) the use of an acousto-optic programmable dispersive filter (AOPDF; Dazzler, Fastlite) installed in our 2.1 μm optical parametric chirped pulse amplifier. Since the phase of RF pulse driving the Dazzler is locked to the mode-locked pulse train, the RF phase can

be transferred to the optical pulse in the process of acousto-optic pulse shaping. Unlike a wedge pair we don't need to add any optical component to change the pump CEP. We actually tested the second method and observed the CEP-dependence of solid-state HHG results which are under further investigations. In either way, changing the signal CEP provides a partialcontrol knob for accessing the CEP of synthesized pulse and also allows presetting the signal CEP to a certain value.

On the other hand, the idler CEP is determined by the relative phase between the signal and pump pulses. Therefore, by adding another thin BaF_2 wedge pair for the pump after the WLG stage to control the relative phase between the pump and signal pulses, we can change the idler CEP without affecting the signal CEP. It should be noted that a precise compensation of delay between the pump and signal pulses is required for not affecting the OPA performance whenever this wedge pair is tuned. This additional wedge pair enables to access and preset the CEP of the idler pulse without changing the signal CEP.

Based on the above arguments, we come up with a procedure of controlling the CEP of combined (synthesized) pulse as follows:

- First, we optimize the OPA to obtain the shortest pulse duration and minimal pedestal (which is the case of current research), by properly adjusting the individual CEP of the signal and idler pulses in addition to the pump-signal delay. Once we find an optimal condition (say, "preset" condition), we do not change the individual CEP of signal and idler pulses.
- Second, we shift the CEP of the combined pulse using a CsI wedge pair, as shown in Supplementary Fig. 10, for the application experiments like HHG.

In summary, we show that the CEP control of the demonstrated pulse synthesis system is feasible.

Supplementary References

1. Lang T., Harth A., Matyschok J., Binhammer T., Schultze M. & Morgner U., “Impact of temporal, spatial and cascaded effects on the pulse formation in ultra-broadband parametric amplfiers,” Opt. Express **21**, 949-959 (2013).
2. Schunemann P. G. & Zawilski K. T., “Large aperture CSP for high-energy mid-infrared generation,” OSA Congress on High-Brightness Sources and Light-Driven Interactions (EUV, HILAS, MICS) (Long Beach, CA, Mar. 20-22, 2016) MS2C.1.
